# Transitions in high-Arctic vegetation growth patterns and ecosystem productivity tracked with automated cameras from 2000 to 2013

**DOI:** 10.1007/s13280-016-0864-8

**Published:** 2017-01-23

**Authors:** Andreas Westergaard-Nielsen, Magnus Lund, Stine Højlund Pedersen, Niels Martin Schmidt, Stephen Klosterman, Jakob Abermann, Birger Ulf Hansen

**Affiliations:** 1 Department of Geosciences and Natural Resource Management, University of Copenhagen, Oestervoldgade 10, 1350 Copenhagen, Denmark; 2 Center for Permafrost (CENPERM), University of Copenhagen, Oestervoldgade 10, 1350 Copenhagen, Denmark; 3 Department for Bioscience, Arctic Research Centre, Aarhus University, Frederiksborgvej 399, 4000 Roskilde, Denmark; 4 Department of Organismic and Evolutionary Biology, Harvard University, Cambridge, MA 02138 USA; 5 Greenland Survey, Asiaq, Svend Jungep Aqqutaa 8, 3900 Nuuk, Greenland

**Keywords:** High-Arctic, Photography, Primary productivity, Time lapse, Vegetation phenology

## Abstract

Climate-induced changes in vegetation phenology at northern latitudes are still poorly understood. Continued monitoring and research are therefore needed to improve the understanding of abiotic drivers. Here we used 14 years of time lapse imagery and climate data from high-Arctic Northeast Greenland to assess the seasonal response of a dwarf shrub heath, grassland, and fen, to inter-annual variation in snow-cover, soil moisture, and air and soil temperatures. A late snow melt and start of growing season is counterbalanced by a fast greenup and a tendency to higher peak greenness values. Snow water equivalents and soil moisture explained up to 77 % of growing season duration and senescence phase, highlighting that water availability is a prominent driver in the heath site, rather than temperatures. We found a significant advance in the start of spring by 10 days and in the end of fall by 11 days, resulting in an unchanged growing season length. Vegetation greenness, derived from the imagery, was correlated to primary productivity, showing that the imagery holds valuable information on vegetation productivity.

**Electronic supplementary material:**

The online version of this article (doi:10.1007/s13280-016-0864-8) contains supplementary material, which is available to authorized users.

## Introduction

Vegetation growth and phenology are important indicators of climate change on both plant level (Cleland et al. 2012) and global scale (Walther 2010). Significant shifts in the timing of annual phenological events have been reported in monitoring studies based on satellite data (Jeong et al. 2011), as well as in in situ observation data on flowering and growing season length (Kerby and Post 2013). Such shifts in seasonality, and the duration of the individual seasons, can have important consequences for the functioning of ecosystems and ultimately on the carbon cycle (McGuire et al. 2009). Understanding the seasonality in relation to climate can thus be a key to an improved understanding of ecosystem response to a warmer climate (Richardson et al. 2013), including biologically driven fluxes of greenhouse gases (Menzel 2002).

Several studies have found Arctic ecosystems to be particularly sensitive to shifts in air temperature (Hinzman et al. 2005; Post et al. 2009), which again influence the vegetation functioning and phenology (Oberbauer et al. 2013; Høye et al. 2013). During the last two decades, vegetation phenology in the Arctic has been monitored using both in situ field measurements focusing on seasonal dynamics in growth (Ellebjerg et al. 2008; Michelsen et al. 2012) and its linkage to CO_2_ exchange (Kross et al. 2014). In parallel, Arctic vegetation has been monitored from satellites (e.g., Zeng et al. 2011), allowing for regional-scale studies. Regional studies report an increase in growing season length from both an advancement of the start of the growing season and a postponed senescence (Zeng et al. 2011), and an increased productivity both regionally (Walker et al. 2006) and locally (Tagesson et al. 2012), although the regional greenness has recently been reported to be declining during 2010 to 2013 (Epstein et al. 2015). However, a meta-analysis by Oberbauer et al. (2013) did not find a general advancement of the start of the growing season based on studies from 12 different Arctic and alpine sites. In addition, it is still uncertain how the relationship between warming and vegetation greening in the Arctic is affected by other variables such as water and nutrients (Xu et al. 2013). Thus, how vegetation growth and ecosystem productivity respond to climate seems highly variable and needs to be addressed at several scales.

The use of repeat photography to detect seasonal variations at a sufficient spatiotemporal resolution has become an increasingly applied tool, also in systems with high spatial heterogeneity such as the alpine and Arctic regions. The technique has been successful in capturing snow-cover fraction and distribution (Bernard et al. 2013), phenology in a wide variety of ecosystems based on a vegetation greenness index (Graham et al. 2010) and a combination of both (Buus-Hinkler et al. 2006; Ide and Oguma 2013). Moreover, derived vegetation greenness from camera images may serve as a good proxy for ecosystem productivity (Westergaard-Nielsen et al. 2013). Consequently, digital cameras provide valuable high spatiotemporal resolution data in the assessment of ecosystem responses to climatic changes.

In this study, we evaluate the phenological response, expressed as the timing of transitions and durations in vegetation greenness, of three high-Arctic plant communities in relation to snow-cover and temperature. We also evaluate the relationship between vegetation greenness and ecosystem productivity over several growing seasons. The study is based on a time series of digital camera images, eddy covariance measurements, and in situ climate data (air and soil temperature, soil moisture, density and depth of snow) from high-Arctic Zackenberg during the years 2000–2013. We hypothesize that snow-cover fraction and end-of-winter snow water equivalents (SWEs), both directly and indirectly, are dominant drivers for seasonal shifts in vegetation growth, followed by temperature changes. We also hypothesize that plant communities responds to inter-annual variation in ambient weather conditions by adjusting growing season length and activity (derived from vegetation greenness) during the start and peak of the growing season rather than during late season.

## Materials and Methods

### Site description

**Fig. 1:**
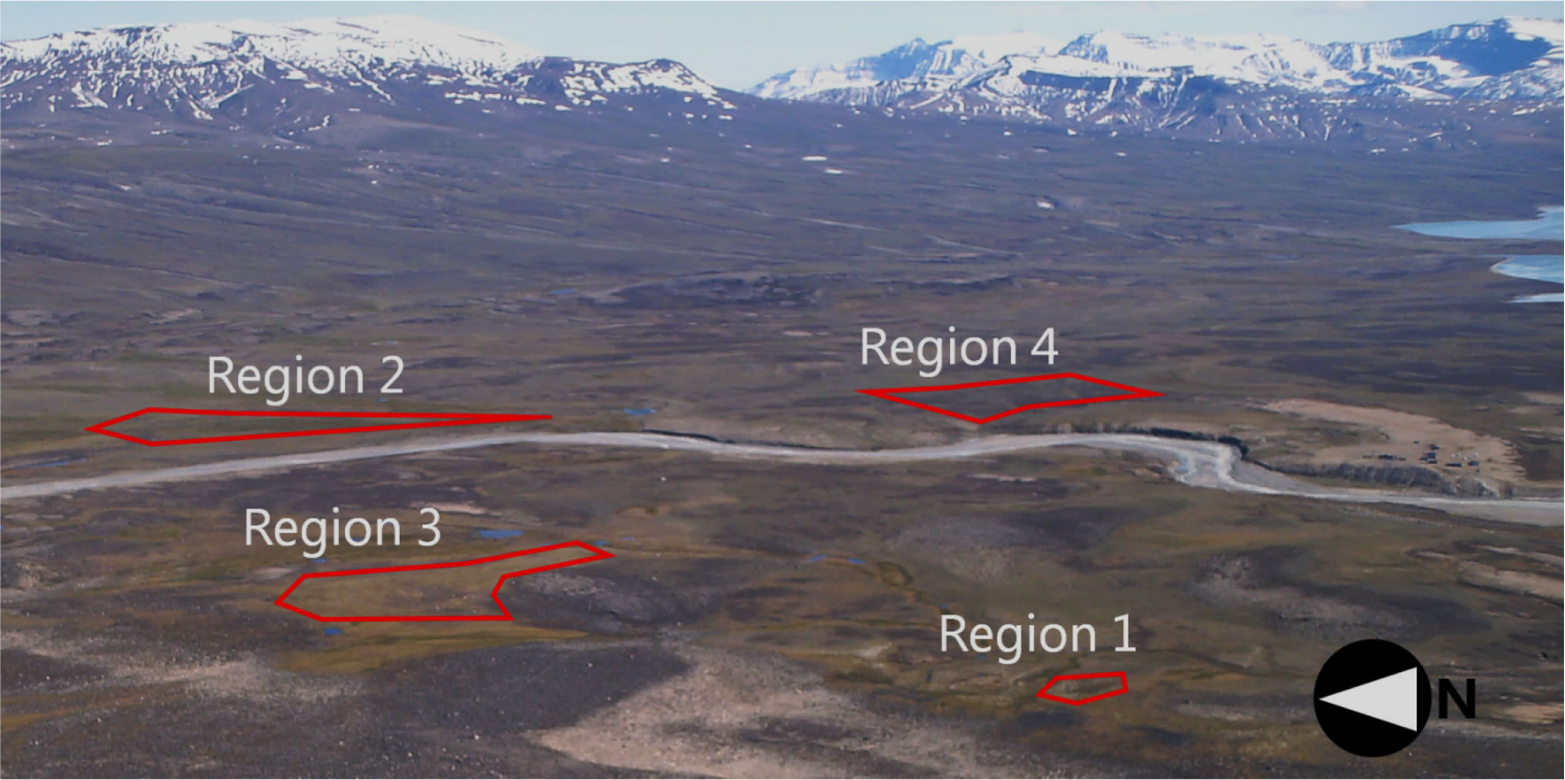
Field of view of the camera located at Zackenberg in high-Arctic Greenland. The regions of interest were selected to capture three dominating plant communities and regions with variation in the timing of the respective snow-free dates. The area is situated approximately 500 m north of Young Sound

The Zackenberg valley is situated at 74°28′N, 20°34′W, Northeast Greenland. The surroundings are mountainous, extending from Zackenberg Mountain at 1400 meters above sea level (m a.s.l.) to slopes and cliffs between 800 and 200 m a.s.l. Below 200 m a.s.l., the topography is flatter and cut through by former and existing rivers (Fig. 1). The Zackenberg valley covers an area of 30 km^2^ on the north side of Young Sound. The majority of vegetation grows in the flat valley below 200 m a.s.l. Despite the proximity to the Greenland Sea, the climate can appear continental with low humidity and high temperature fluctuations due to the build-up of sea ice. Annual mean temperature (1996–2014) is approximately −9.0 °C with July as the warmest month (6.3 °C) and February as the coldest at −19.8 °C (Jensen and Rasch 2014). The annual precipitation is <250 mm, of which approximately 85 % falls as snow. Maximum snow depth, measured from 1997–2014 by automated sensors in the valley, varies from 0.13 m (winter 2012/2013) to 1.44 m (winter 2014/2015). The spatial distribution of snow is consistent from year to year due to a predominant wind direction from the North during winter (Hansen et al. 2008). Long-term monitoring data from Zackenberg are available through the extensive cross-disciplinary ecological monitoring program (ZERO, www.zackenberg.dk) by Greenland Ecosystem Monitoring (GEM).

The study is focused on four regions in the lower part of the Zackenberg valley within the field of view of a single camera (see Table 1; Fig. 1). Within the camera field of view of approximately 15 km^2^, *Cassiope*-dominated heath, *Dryas*-dominated heath, and fens with *Eriophorum* cover 10–14 % each. Grasslands dominated by graminoids cover up to 40 % and snow beds (with deep late-winter snow depths) with *Salix*, *Vaccinium*, and *Alopecurus*, cover 20 % (Elberling et al. 2008). The four regions were thus demarcated from a rationale to capture a variety of the most abundant plant communities in Zackenberg as well as a representation of plant communities growing in low to medium and high soil moisture, respectively, to capture possible variability across a moisture gradient (Bay 1998). Moreover, two fen areas were included to assess the effects of differences in the timing of snowmelt resulting from differences in winter snow accumulation.

**Table 1:** Overview of the studied regions. Average snow-free date is expressed as average day of year (DOY) with <20 % of the surface covered with snow. Average date was based on data from 2000 to 2013. Vegetation classifications were conducted as quadrat point analyses following the ITEX standard (Bay 1998)

ROI	Characteristics
High soil moisture	
1	Fen dominated by *Arctagrostis, Eriophorum scheuchzeri, Polygonum* spp, and mosses. Average snow-free date: DOY 157
2	Fen dominated by *E.scheuchzeri, Carex stans*, and *Dupontiapsilosantha*. Average snow-free date: DOY 169
Medium–low soil moisture	
3	Grassland dominated by *C. bigelowii*, *C. capillaris*, and *E. triste*. Spread *Salix arctica* individuals. Average snow-free date: DOY 166
4	Heath dominated by *Cassiope tetragona*, *S. arctica*, and *Vaccinium uliginosum*. Average snow-free date: DOY 170

Water availability in the growing season is expected to impact the growing season dynamics due to the limited precipitation in Zackenberg. Here we used annual SWE and soil moisture as descriptors of water availability for the whole-growing season since the pre-melt snow pack is the main contributor of water through the growing season (Hansen et al. 2008). Continuous measurements of SWE and soil moisture conditions were only available for region 4.

### Image data and processing

#### Cameras

RGB (red–green–blue) multispectral broad band data were obtained from digital cameras mounted at 400 m a.s.l. on an east-facing slope of the Zackenberg Mountain. The cameras overlooking the valley were mounted with a horizontal tilt angle of 15° with varying pixel resolutions (Table S1). The cameras obtained a daily image at noon, local winter time (UTC), covering an area of approximately 15 km^2^ (Table S1). Images from camera 1 were converted from a native KDC format to JPG using reaConverter (reasoft). Images from cameras 2 and 3 were natively stored as JPG by the camera firmware, as this was the only option. All JPG images used the standard 24-bit format, i.e., 8 bits per color channel with digital numbers (DNs) from 0 to 255. Replacement camera systems were installed continuously when malfunction occurred, as well as for improved image quality. All systems were mounted in weatherproof boxes with a window of low-iron glass to avoid a green cast and powered by batteries and solar panels.

#### Processing

**Fig. 2:**
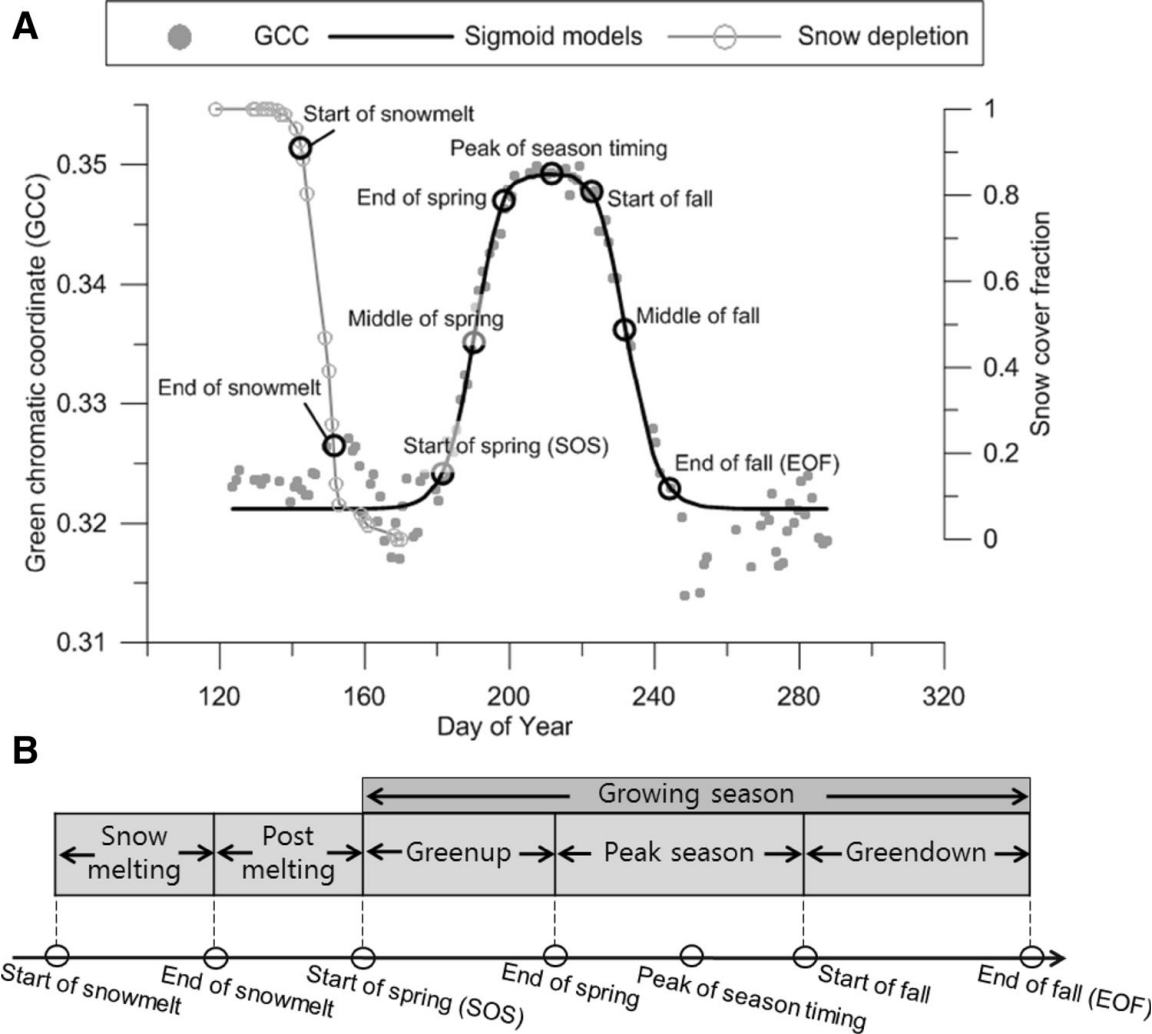
**a** Conceptual plot of a fitted double sigmoid model and the corresponding transitions dates. Also depicted is snow-cover fraction, including the *start of snowmelt* and *end of snowmelt*. **b** Durations between transition dates and corresponding denotation. *Snow melting* = end of snowmelt − start of snowmelt; *Post-melting* = start of spring − end of snowmelt; *Greenup* = end of spring − start of spring; *Peak season* = start of fall − end of spring; *Greendown* = end of fall − start of fall

To detect snow and vegetation greenness, the annual time series of images were manually looked through and filtered for low-quality data (winter darkness, fog, precipitation, and heavy shadows), resulting in an average of 71 images per growing season covering a period from late May to mid-September. Year 2005 was omitted due to severe data gaps resulting from camera malfunction. The images were processed in a custom Matlab (MathWorks Inc.) script which imported the JPG images as individual RGB channels using the built-in image import tool. The regions were demarcated by manually defined masks. Within the masks, pixels with snow were classified using a dynamic threshold (Salvatori et al. 2011) based on average gray level DN from all RGB channels ((R + G+B)/3). The threshold function finds local histogram minima using the Matlab “findpeaks” function. For images without an automatically detectable threshold, we used a manually set base threshold. The initiation of snowmelt was set as the day of year (DOY) with 90 % snow-cover fraction in a region; it was considered snow-free when less than 20 % of the pixels were classified as snow-covered. Accordingly, the DOY for less than 20 % was denoted end of snowmelt (Fig. 2a, b). Pixels classified as snow-covered were subsequently masked away. Vegetation greenness was computed for pixels not covered by snow as the green chromatic coordinate (GCC):

1 $${\rm{GCC }} = {\rm{ }}{{\rm{G}}_{{\rm{DN}}}}/({{{\rm{R}}_{{\rm{DN}}}} + {\rm{ }}{{\rm{G}}_{{\rm{DN}}}} + {\rm{ }}{{\rm{B}}_{{\rm{DN}}}}}),$$

where DN is the digital number describing the 8-bit gray level intensity in a range from 0 to 255, and *R*, *G*, and *B* correspond to the red, green, and blue spectral bands, respectively. The GCC has been shown to correlate with NDVI in time and space (Richardson et al. 2007; Westergaard-Nielsen et al. 2013) and time series of GCC has consequently been used in an increasing number of studies of temporal transitions in plant phenology (e.g., Ide and Oguma 2013; Sonnentag et al. 2012). Time series of snow-cover fraction and GCC data were then used for further analyses.

#### Estimating transition dates

Various methods have been proposed to derive dates for significant shifts in data time series describing vegetation phenology, including absolute thresholds, derivatives, model fit, and transformations (de Beurs and Henebry 2010). In this study, the relative and not absolute GCC data, produced from different cameras, preclude the use of fixed thresholds because different cameras respond differently to the visible wavelengths of light. Nevertheless, Sonnentag et al. (2012) found an acceptable error between camera models when extracting transition dates as relative points based on the curvature of a model fitted to the time series of GCC data. Earlier studies have suggested the use of second-order polynomials when modeling high-Arctic vegetation greenness (Buus-Hinkler et al. 2006; Tamstorf et al. 2007); however, such models fail to describe potential differences in the duration of greenup and senescence, i.e., skewed phases. Sigmoid fit models offer a better representation of such differences, while still utilizing all possible data (Zhang et al. 2003). Moreover, they have been found to be robust to the addition of random noise (Fisher et al. 2006). Transition dates were therefore derived from double sigmoid models fitted to the beginning and end of each growing season, respectively. Each sigmoid model can be expressed as follows:

2 $$ y\left( t \right) \, = \, \left( {c \, /{ 1} + {\text{e}}^{{{\text{a}} + {\text{bt}}}} } \right) + d, $$

where *t* is the time; *a* and *b* are the parameters determining the timing of curvature increase or decrease; c is the amplitude; and d is the initial value of *y*. Six dates describing *start of spring*, *middle of spring*, *end of spring*, *start of fall*, *middle of fall*, and finally *end of fall* were identified by analyzing the rate of change of curvature in the sigmoid models (Fig. 2a; Klosterman et al. 2014):

3 $$k = {\mkern 1mu} \left( {{f^{\prime \prime }}\left( t \right)} \right){\mkern 1mu} /{\mkern 1mu} {\left( {{{( {1 + \left( {{f^\prime }\left( t \right)} \right)} )}^2}} \right)^{(3/2)}}$$

where *k* is the curvature and *f* are the sigmoid functions at time step *t*. In addition, the peak of season timing was defined as the time of maximum GCC from the curve fit. If the maximum GCC occurred for more than one day, the average time was selected to represent peak of season timing. The sensitivity of the transition dates was evaluated using 100 Monte Carlo samplings of the model parameters (using Matlab) of the fitted sigmoid functions. The inner 90 % range was then used as confidence intervals; see Klosterman et al. (2014).

### Climate and monitoring data

#### Climate data

Robust year-round precipitation data in the studied period are scarce in Zackenberg. However, the majority of the precipitation falls as snow, and we therefore estimated the annual SWE as the water available for plant growth assuming no prior snowmelt. SWE was calculated from manually collected snow densities and snow depth. Snow density (g cm^−3^) samples were collected in the period from maximum snow depth until onset of spring snowmelt in May–June during the years 2004–2013. The annual end-of-winter snow bulk density was estimated from metal tube samples of 20 cm height in snow pits dug on the heath-covered valley floor in proximity to the main climate station (74°28′20″, 20°33′08″, 38 m a.s.l.) or samples made with a Standard Federal Snow Sampler (Clyde 1932) in the same area. The snow depth used in the SWE calculation was derived from total depth of the snow pit or the length of the snow sample/core in the Snow Sampler. SWE was determined as the product of the snow bulk density and snow depth divided by the density of water. The sample size of the annual end-of-winter SWE estimations ranges from 1 to 6 observations (Pedersen et al. 2016). SWEs in 2000–2003 were extrapolated from measured end-of-winter snow depths from an automated snow-depth sensor at the main climate station, using a linear model fit between SWE and snow depths based on observations made in 2004–2013.

Air temperatures were measured 2 m above terrain level at hourly frequency at the main climate station, and were expected to be representative for all the analyzed regions since they are located at similar elevations and within a maximum of 1.5 km from the climate station. Soil temperature data at 5 cm depth were available from 2000 to 2013, measured at a *Salix*-*/Cassiope*-dominated heath near the main climate station. Soil moisture data at 10 cm depth were available from 2004 to 2013 as bi-weekly measurements of volumetric water content from a mixed heath site in proximity to region 4. Incoming shortwave radiation at hourly frequency from 2000 to 2011 is likewise expected to be representative for all regions. Exact instrumentation and protocols for temperature logging, soil moisture measurements, and radiation measurements are described in the annual ZERO reports.^1^ The area west of the Zackenberg River is not represented directly with soil temperature and moisture, as the area has been impossible to access frequently due to the high baseflow in the river. Instead, camera data are available allowing for the inclusion of areas west of the river in this study.

#### Ecosystem productivity

Measurements of the net ecosystem exchange of CO_2_ (NEE) using the eddy covariance technique have been conducted since 2000 at the *Cassiope*-dominated heath site in Zackenberg, i.e., within region 4. Until 2007, the eddy covariance system consisted of a closed path infrared gas analyzer LI-6262 (Li-Cor Inc., USA) and a 3D sonic anemometer Gill R2 (Gill Instruments Ltd., UK). In the autumn 2007, the instrumentation was upgraded to a LI-7000 (Li-Cor Inc., USA) and a Gill R3 (Gill Instruments Ltd., UK). The two systems were running in parallel during two autumn months, and no significant differences were found between the obtained CO_2_ fluxes. Gross primary production (GPP) was derived from the NEE measurements using a light response curve approach. Details on the eddy covariance measurements, data processing, flux footprint, and flux partitioning can be found in Lund et al. (2012). Modeled daily averages of GPP were subsequently regressed on daily GCC within region 4 to link the camera imagery to quantified in situ measurements of ecosystem productivity.

#### Statistics

Statistical analyses of correlations and trends between the growing season (as inferred from the camera imagery) and the ambient biotic and abiotic conditions were computed with the Statistical Analysis System (SAS Institute), using ordinary least squares linear regressions and generalized linear models.

## Results

The terminology in this section is based on Fig. 2a, b. An event occurring relatively earlier in time is denoted as an advance. Events occurring relatively later in time are referred to as a postponement.

### Snow-cover

**Fig. 3:**
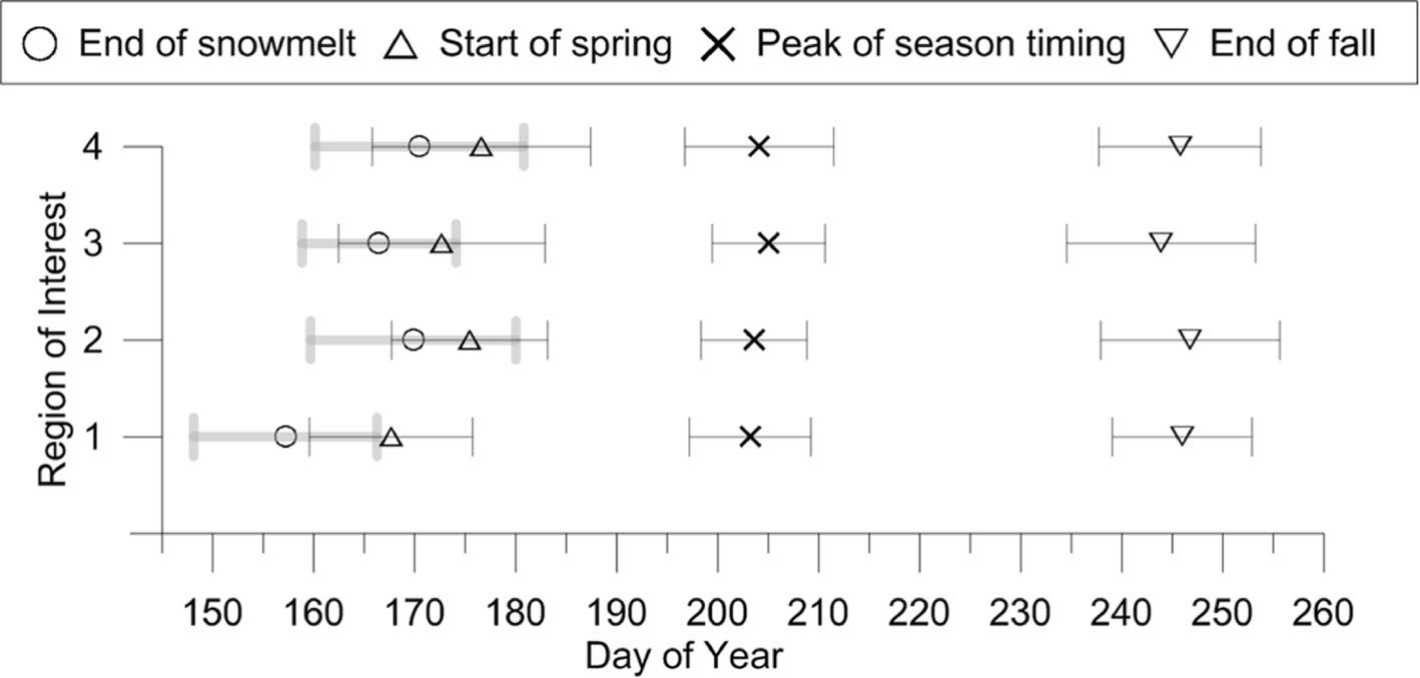
Average transition dates in the period 2000-2013 for the four regions. Error bars showing standard deviation of the transition date over the 13 measured years

There was no significant advancement of end of snowmelt during the studied period. We did not, however, linearly correct for leap years, meaning that an uncertainty could theoretically be resulting from this. The longest snowmelt duration (i.e., days from 90 % to <20 % snow coverage) was seen for regions 1, 2, and 4 (average of 9.5 days, SD ± 4.1), and the shortest for region 3 (average of 7.5 days, SD ± 1.9). Region 1 (i.e., early snow-free fen) had the longest post-melting duration on average, equaling 11 days (Fig. 3; Table 2). In contrast, the shortest post-melting duration was seen for the other fens, region 2.

**Table 2:** Linear ordinary least squares regression between end of snowmelt (<20 % snow-cover) and start of spring (SOS). ‘End of snowmelt to SOS’ refers to days between the two variables. All correlations were statistically significant with a *P* value < 0.005

Region	*R* ^2^	Slope	RMSE	End of snowmelt to SOS in days	Stdev. of End of snowmelt to SOS
1	0.60	0.68	5.32	10.5	5.86
2	0.79	0.67	3.74	5.6	4.89
3	0.84	1.23	4.22	6.2	4.41
4	0.92	1.00	3.19	6.1	3.05

### Phenological transitions

The biotic transition dates are defined as start of spring (SOS), middle of spring, end of spring, peak of season timing, start of fall, middle of fall, end of fall (EOF); see Fig. 2b. The 90 % confidence interval of the transition dates resulting from the Monte Carlo samplings were on average +3.5 and −3.6 days, i.e., the sensitivity was a week for all transition dates on average. SOS dates were estimated within +3.9 and −2.4 days, and EOF within +4.4 and −6.0 days.

SOS, i.e., start of spring (Fig. 2b), occurred between DOY 154 and 190 with an average of DOY 173 (22 June, SD ± 9.7) for all the regions. EOF was on average 73 days after SOS (2 September, SD ± 8.2), ranging from DOY 226 to 265. Peak of season timing occurred between DOY 192 and 220. The earliest peak of season timing for all regions was observed in year 2013, which had very limited snowfall and consequently a dry growing season. Interestingly, the SD for the timing of the peak season was the lowest for the transition dates illustrated in Fig. 3, followed by EOF. End of snowmelt and SOS showed the greatest variation both in time and space of the illustrated transition dates.

There was a statistically significant advancement of SOS of 10 days averaged for all regions (*R* ^2^ = 0.10; *P* = 0.021) during the study period, with no significant difference between the regions (*p* = 0.84). The EOF also advanced at a similar rate during the 14-year period (11 days on average, *R* ^2^ = 0.19; *P* = 0.0012), with no difference between the regions. The net result was consequently an unchanged growing season length. The longest greenup duration, i.e., days between SOS and end of spring (Fig. 2b), was observed for region 1 (fen with early snowmelt). The longest peak season duration, i.e., days between end of spring and start of fall, was seen for the driest region 4. Regions 3 and 4 had the lowest peak GCC on average, resulting from a flatter peak season period as modeled by the sigmoid functions. Hence, the plant communities in regions 3 and 4 have species with lower greenness (and in region 4, more consistent greenness). Average greendown duration for the regions (the period from start of fall to EOF) was 27 days for regions 3 and 4, and four to five days longer in regions 1 and 2 (Fig. S1).

#### Snow-cover, soil moisture, and transition dates

**Fig. 4:**
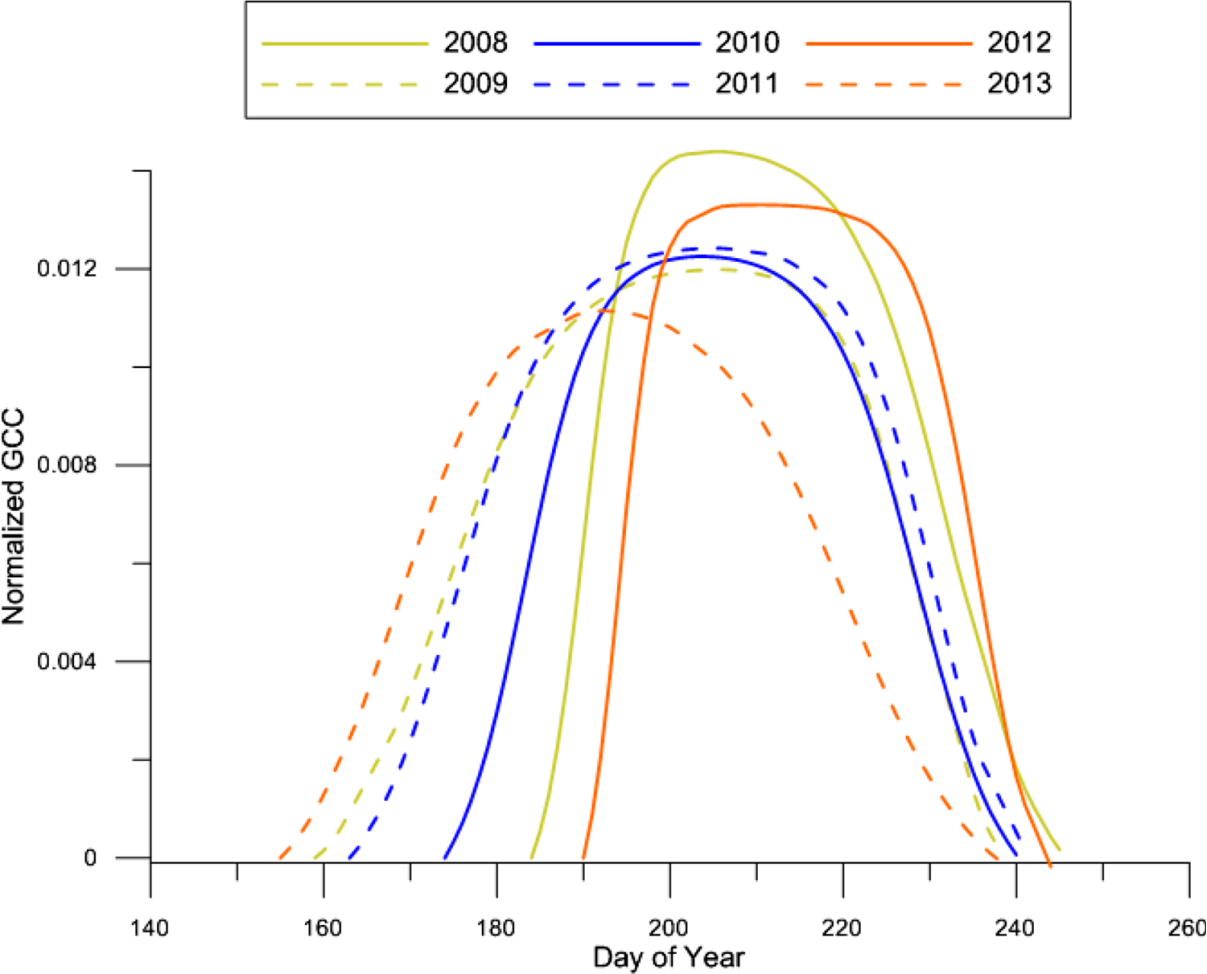
Temporal modeling of GCC from 2008 to 2013 in region 4. GCC values are normalized to GCC at start of season. Data are based on the same camera model to allow direct comparison

We found the timing of end of snowmelt to be significantly correlated with SOS (*P* < 0.005), (Table 2) and EOF (Table 3). The correlations were significantly different between the regions (Table 3, see result for region*end of snowmelt, regarding SOS). Generally, a late end of snowmelt resulted in short greenup durations (e.g., year 2002) and relatively longer greendown durations (Fig. S2). Years with late snowmelt, and consequently late SOS, usually had late EOF. The relatively snow rich years 2008, 2010, and 2012 all had later EOF compared to the years 2009, 2011, and 2013 with less snow (Fig. 4).

**Table 3:** Generalized linear model analysis of correlation between (1) end of snowmelt/start of spring (SOS), and end of snowmelt/end of fall (EOF), respectively, and (2) greenup duration and greendown duration response to the predictors SOS, EOF, and SWE. Responses of greenup and greendown durations are reported both for region 4 only and all regions (results from all regions are shown in parenthesis). There were statistically significant differences between the regions regarding the greenup duration, whereas we found no significant effect of the interactions region*SOS, region*EOF, SWE*SOS, or SWE*EOF

Predictor	Mean sq error	*P* value	Predictor	Mean sq error	*P* value
End of snowmelt and SOS			End of snowmelt and EOF		
End of snowmelt	3734	<0.001	End of snowmelt	1224	<0.001
Region*End of snowmelt	66	=0.017	Region*End of snowmelt	–	=0.85
Greenup duration			Greendown duration		
SWE	264 (710)	=0.002 (<0.001)	SWE	366 (223)	=0.026 (0.014)
SOS	231 (1248)	=0.003 (<0.001)	EOF	2227	=0.004 (< 0.001)
Region	–	(=0.51)	Region	–	(=0.43)

**Fig. 5:**
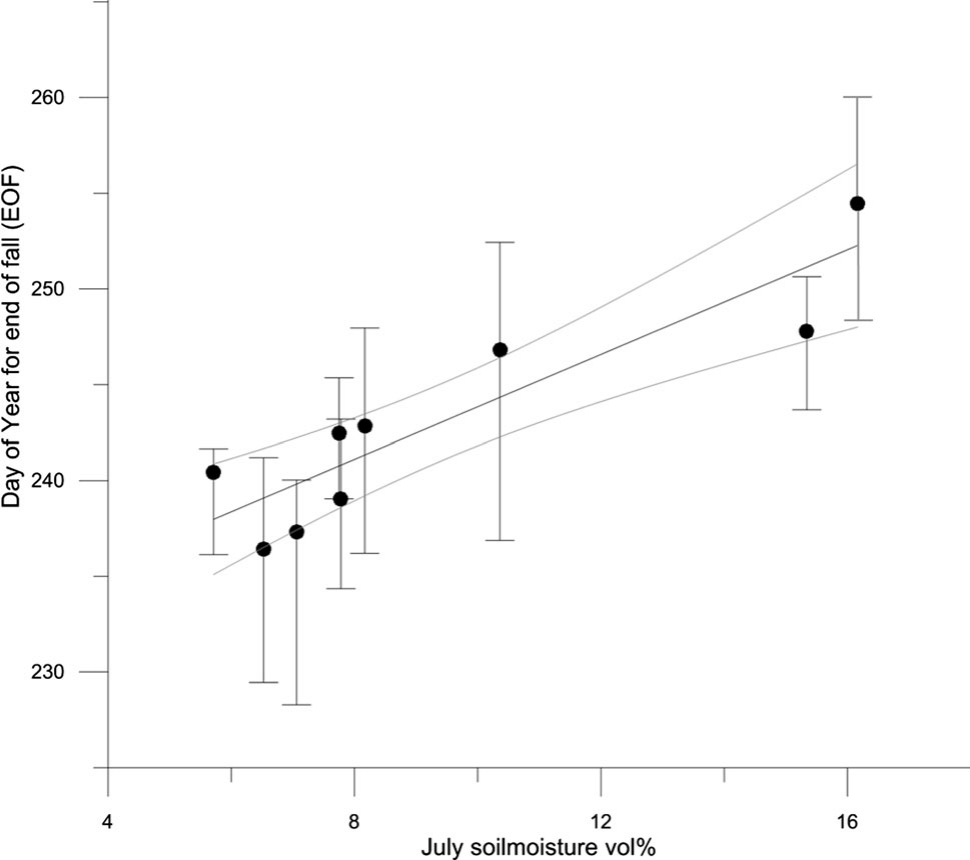
Timing of end of fall (EOF) plotted against July soil moisture in ROI 4. Gray curved lines are 95 % confidence intervals. *Bars* indicate EOF timing sensitivity based on the Monte Carlo samples

We correlated the average soil moisture vol% from 10 cm depth in July with EOF and greendown duration, respectively, in region 4, to evaluate the effect of soil moisture during mid-growing season on the late season vegetation growth. The timing of EOF was significantly correlated to the average July soil moisture (*R* ^2^ = 0.77; *P* = 0.002, Fig. 5) as well as with the greendown duration (i.e., period between start of fall and EOF) (*R* ^2^ = 0.45, *P* = 0.047). Consequently, years with moist July conditions have longer senescence phases and a later end of fall, and vice versa. The soil moisture isolated did not explain the growing season duration or the peak value in GCC (of which the latter was normalized to values between 0 and 1 to allow comparison between cameras). However, a combination of SWE and July soil moisture explained 72 % of variations in growing season duration (*R* ^2^ = 0.72, *P* = 0.022).

#### Temperature and transition dates

Below-zero temperatures in the autumn were expected to accelerate senescence, and thus affect the duration of the greendown period. However, we did not find a correlation between degree days freeze (i.e., the sum of daily mean temperatures below 0 °C) in the autumn months August/September and greendown duration. The timing of the first occurrence of daily air temperature averages below 0 °C in autumn was not correlated to EOF or greendown duration either. Likewise, we did not find degree days thaw (i.e., the sum of daily mean temperatures above 0 °C) during May–July or the summer warmth index (i.e., the sum of monthly average air temperatures above 0 °C) to be explanatory variables for SOS or the greenup duration. In contrast, average soil temperature in June measured in region 4 was significantly correlated to the duration of greenup in the same region (*R* ^2^ = 0.56; *P* = 0.008), indicating that higher soil temperatures coincided with longer greenup duration, although this is likely a secondary effect of snow rich years having higher soil temperatures in early season due the insulating snow. The duration of greendown (i.e., senescence) or timing of EOF was not correlated to average soil temperatures in the corresponding period.

### Ecosystem productivity

The shift from positive to negative NEE flux (i.e., the timing in early growing season when the system switches from functioning as a net source to a net sink of atmospheric CO_2_ on a daily basis) in region 4 occurred on average at DOY 178, whereas the shift from negative to positive NEE in late growing season occurred at DOY 229 in the studied period (2000–2013). Consequently, the source to sink shift date was also significantly correlated with SOS (Table 4). On average, EOF occurred 7 days later than the shift from sink to source was measured, but they were not correlated.

**Table 4:** Results of ordinary least squares linear regressions. The ecosystem switch from source to sink for atmospheric CO_2_ (NEE-spring) was significantly correlated to end of snowmelt and thereby start of spring (SOS). A statistically significant relationship was not found for end of fall (EOF) and the shift back to CO_2_ source in autumn (NEE-senescence)

Correlated variables	*R* ^2^	Slope	Intercept	RMSE	*P* value
End of snowmelt/NEE-spring	0.85	0.844	35.2	12.6	<0.001
SOS/NEE-spring	0.75	1.0	5.7	5.6	<0.001
EOF/NEE-senescence	0.13	0.29	167.9	6.3	=0.22

The link between vegetation greenness and actual ecosystem productivity was validated against modeled daily average GPP data based on NEE. We found GCC from region 4 (the region representing the eddy covariance fetch) to be a highly significant predictor for GPP, with a significantly different relationship depending on camera model (Table S2). This means that the correlation model between GCC and GPP is unique for a given camera.

**Fig. 6:**
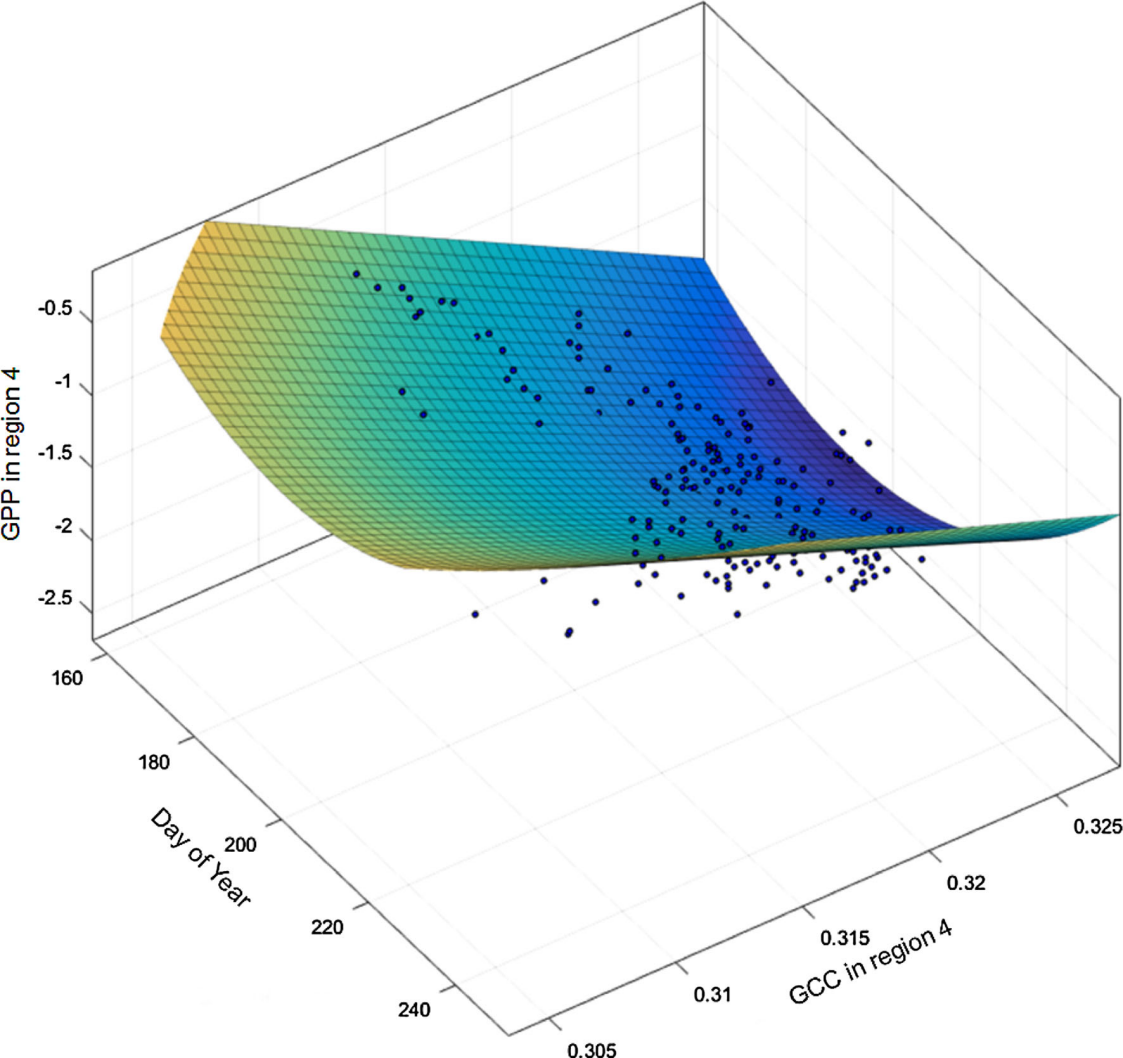
Three-dimensional correlation model of GPP, GCC, and Day of Year. The model is based on a second-order polynomial in the X-plane (time) and a first-order in the GPP/GCC plane, and expressed as *f(x, y)* = *12.82* − 0.1347*x + 18.77*y + 0.00052*x ^*2*^ − 0.2198*x*y. The model fit was statistically significant (adjusted *R* ^2^ = 0.71, *P* < 0.001, RMSE = 0.28)

The statistics from Table S2 allowed us to fit a three-dimensional polynomial model of GCC and GPP during the growing season, to evaluate the possible seasonal biases in the correlation between GPP and GCC as a function of DOY. Due to the effect of the camera type, we based the model on data from camera type 3 (see Table S1). The model fit improved significantly when adding a second order in the temporal dimension, while adding a second order in the GPP-GCC dimension did not improve the model fit compared to a first-order linear polynomial (Fig. 6).

## Discussion

### Region-specific variations

The highly significant correlation between end of snowmelt and SOS is consistent with earlier studies on Arctic ecosystems (Mastepanov et al. 2013) and NDVI measurements of greenup (Buus-Hinkler et al. 2006). The faster greenup durations seen in regions 2 and 4 are likely a combination of deeper snowpacks and higher spring soil temperatures (average increase from −0.9 to 2.8 °C in 5 cm in the same period) and air temperature (average increase from 2.2 to 3.4 °C in the same period) during the snow-free period. Indeed, the correlation between average soil temperatures in June and SOS underlines this argument. Higher soil temperatures during the start of the growing season, resulting from the preceding winter’s snow regime, will also increase nutrient availability (Johansson et al. 2013) which could also decrease the greenup duration. Long greenup durations, as seen in region 1, could accordingly be due to low soil temperatures (caused by thin, less insulating snow-cover in winter) prohibiting an immediate greenup after snowmelt (Lipiec 1990). The model slopes in Table 2 indicate that a later end of snowmelt means a shorter post-melting duration (time between end of snowmelt and SOS) in regions 1 and 2 (fen), a stable duration in region 4 (heath), regardless the timing of end of snowmelt, and an slightly increasing post-melting duration with a late end of snowmelt in region 3 (grassland). This is likely resulting from differences in the greenness build-up during spring of the different plant communities (e.g., proportion of mosses and vascular plants), as well as generally shallower snow depths in a gradient from fen to grassland to heath, which will affect the soil temperature regime during spring.

The lower peak GCC and longer peak season duration in the drier regions is in agreement with earlier NDVI measurements of similar plant communities (Ellebjerg et al. 2008), and short greendown durations in these regions indicates a faster senescence due to limited water availability in late season rather than light or temperature. This argument is supported by the correlation between July soil moisture and both EOF and greendown duration, and show that soil moisture holds potential as a predictor for the timing of EOF. This is relevant since the literature has reported EOF to be challenging to find proxies for (Cleland et al. 2007). As soil moisture is closely linked to SWE, the relationship between soil moisture and EOF is arguably valid for all the regions. The lack of a correlation between phenological transition dates in the fall and temperature variables likewise suggests that a general atmospheric warming may not prolong the growing season markedly because it is rather limited by incoming solar radiation and water availability. Other key phenological events such as flowering duration and interactions with pollinators can, however, be accelerated from a warmer climate in Arctic ecosystems (Schmidt et al. 2016).

The general advancement in SOS (least pronounced in region 1) was mainly related to an earlier end of snowmelt. It may be a consequence of elevated spring air temperatures (Euskirchen et al. 2006; Menzel et al. 2006) or a consequence of a decrease in spring snow-cover in Zackenberg (Pedersen et al. 2016). Since the timing of the first spring thawing temperature sets the threshold to the earliest possible advancement in end of snowmelt (Chapin 1983), increased spring temperatures are likely to have the largest effect on the regions with late snowmelt.

### Inter-annual variation

The inter-annual variability in snow-cover is marked, which is in agreement with the reported observations by Pedersen et al. (2016). This variability seems to affect the whole-growing season, with an increasing effect in early growing season. The seasonal patterns in GCC in the dry regions 4 and 3 had a tendency to offset a late greenup with a relatively higher peak GCC value, while EOF had little variance (Fig. 4). Moreover, the growing season duration was significantly longer in years with high SWE and soil moisture content in July. This indicates that the represented plant communities are, to a certain extent, adapted to such inter-annual variation in end of snowmelt, which have been present in Zackenberg for several decades (Pedersen et al. 2016), perhaps as a result of their phenotypic plasticity (Totland and Alatalo 2002). From an ecosystem productivity perspective, the ability to offset variation in SOS by increasing peak greenness agrees with Parmentier et al. (2011) who found that timing of snow melt isolated did not correlate with net CO_2_ uptake, but rather the whole-growing season must be considered.

An increase in growing season duration resulting from both an advancement in SOS and delay of EOF has been reported from satellite-based studies of high-latitude phenology (Zeng et al. 2011, 2013). The satellite studies furthermore report a highly heterogeneous pattern across geographical location and sensor type. The advancement in SOS in high latitudes based on the MODIS sensor (Zeng et al. 2011) corresponds with our findings from Zackenberg, whereas a reported delay in EOF of 2.2 days per decade (Zeng et al. 2011) contradicts our findings of a coincident advancement in EOF. As requested in the aforementioned MODIS-based study, this suggests the use of ground-based vegetation index data as a platform for inter-calibration of satellite-derived vegetation indices and phenology metrics.

### GCC and ecosystem productivity

The statistically highly significant correlation between GCC and GPP justifies the use of GCC as a proxy for ecosystem productivity. As earlier reported by Lund et al. (2012), the shift from source to sink is correlated to end of snowmelt (*P* < 0.001). Here we found the source–sink shift for CO_2_ in NEE during spring to be correlated with SOS, which further underpins the applicability of GCC as a proxy for temporal shifts in ecosystem exchange of CO_2_. The relationship between EOF and the autumnal NEE shift from sink to source was not correlated, which we interpret as a result of litter fall and a resulting increase in respiration rates from microbial decomposition of the litter (Mikan et al. 2002). Hence, the shift to a net release of CO_2_ during senescence would appear earlier than the camera-based EOF, in agreement with the findings in our study.

Despite being both site- and camera-specific, the significant fit of the visualized model (Fig. 6) suggests that GCC can be used as an indicator for GPP in very low productive Arctic regions in general. The better model fit obtained by adding a second-order polynomial suggests a variation in the intersect of the linear correlation between GPP and GCC over time. This is likely related to a hysteresis caused by a higher photosynthetic efficiency per leaf area in spring than in late season (Westergaard-Nielsen et al. 2013). Consequently, the establishment of robust 3D-models can improve the potential use of GCC as a site-specific GPP proxy in the studied area.

The link between growing season duration and ecosystem productivity is ambiguous (Euskirchen et al. 2006; Hu et al. 2010), with indications of both decreasing and increasing ecosystem productivity following longer growing seasons. Growing season length can thus not solely be used as an indicator of productivity (Parmentier et al. 2011). However, Xia et al. (2015) suggest a combination of season duration and peak GPP as a robust measure of annual GPP across a number of biomes and disturbances. Following this rationale, camera-derived GCC data has a unique applicability in temporary setups of eddy covariance campaigns with overlapping time series of images. When a robust site-specific model has been established (Fig. 6), camera data can subsequently be used as proxy for ecosystem productivity, which could provide valuable input to an improved understanding of the spatiotemporal variation between GPP and plant phenology. Nevertheless, further research on applied methods to allow direct comparison of GCC from different sites and cameras is urgently needed to expand and improve the scientific value of camera-derived indices in ecosystem monitoring.

### Technical considerations

Using JPG images introduces artifacts related to the image compression and conversion between color spaces. The available data did not offer lossless images, and we chose JPG as this involved the fewest processing steps and thus fewer risks of losing image information. However, using averaged values from regions with >160 pixels will mitigate the risk of introducing significant artifacts, and using JPG has been found not to affect the timing of extracted transition dates (Sonnentag et al. 2012). The use of a dynamic threshold in the binary classification of snow-covered pixels allows for detection of snow, despite changes in scene illumination. However, misclassified pixels are unavoidable. The active layer in the soil can still be frozen immediately after end of snowmelt, resulting in poor drainage of the melt water and thus standing water at the ground surface. From certain illumination angles causing high reflectance in the camera direction, such water-covered pixels could erroneously be classified as snow. Consequently, we conservatively chose the 20 % snow-cover date as end of snowmelt. Following snowmelt, there was a local minimum in the computed GCC before it increased as a result of greenup (Fig. S3). We interpret the GCC dip as being caused by the water-saturated conditions following snowmelt. The moist conditions in a majority of illumination angles appear darker than dry conditions, which can result in lower GCC. A similar phenomenon is known from satellite-derived time series of NDVI, which decrease over very moist surfaces that cause NIR absorption (Farrar et al. 1994).

The influence of scene illumination is apparent in GCC (Sonnentag et al. 2012), and data can be filtered from several daily images to mitigate short-time variations. The available frequency in this study does not allow for daily averaging so we addressed the issue by fitting a smoothing model. Nevertheless, missing data or a number of successive days with poor illumination (e.g., severe reflections or fog) may result in a model bias. We addressed the problem by visually sorting the images and dismissing outlier images. Preferably, this subjective selection should be avoided, yet we did not find automated selection criteria (e.g., Ide and Oguma 2013) to be sufficiently effective. The fitted sigmoid models and the associated computed transition dates are numerically solved, and thus include an uncertainty. We have quantified the uncertainty to be larger at the end of season than seen for the start of season, and with a bias toward an underestimation of the growing season length. Based on the end of season uncertainty, we see no marked differences in the timing of EOF between the four regions. However, SOS seen in region 1 is still earlier than the corresponding dates for regions 2, 3, and 4, when taking the uncertainty into account. In general, the region-specific variations are less pronounced when including the uncertainty, while the annual variation is considerably larger.

Arguably, bi-directional effects are influencing the time series of GCC and must be taken into consideration when comparing the different regions. The low camera angle should preferably have been closer to 45°, to avoid potentially systematic GCC bias between erectophile and planophile plant communities (Jones 1984). At the initial installation of the cameras, the coverage and continuity of the field of view were prioritized instead of camera angle. Ultimately, the fixed position of the cameras allows us to compare the time series inter-annually, but possible biases in absolute GCC values must be considered when comparing data from the regions.

## Conclusions

In this study, we present 14 years of image- and gross primary productivity data from high-Arctic Zackenberg. We show that the green chromatic coordinate vegetation index can successfully be used to determine phenological transitions in vegetation growth in high-Arctic ecosystems. Moreover, we show that timing of snowmelt and end-of-winter snow water equivalents are closely linked to not only spring greenup, but also peak timing and peak green chromatic coordinate value as well as the growing season duration. We find that air temperatures are not the primary explanatory variable for growing season length when based on transition dates from the green chromatic coordinate. In addition, we do not find a significant increase in average growing season length due to a significant average advancement in start of spring of 10 days and end of fall of 11 days. Rather, snow water equivalents and soil moisture were the most limiting factors for plant growth in the studied area, of the analyzed parameters, suggesting that elevated temperatures alone will not prolong the growing season. Finally, we conclude that the green chromatic coordinate is a robust proxy for gross primary productivity across years with considerable climatic variations, and that it, in combination with temporal modeling of seasonal plant phenology, has great potential in estimating plant productivity and ecosystem carbon cycling in low productive high-Arctic ecosystems. However, further research on the derivation of absolute or comparable data across camera models are needed to improve and expand the use of camera-derived vegetation indices in ecosystem monitoring.

## Electronic supplementary material

Below is the link to the electronic supplementary material.

Supplementary material 1 (PDF 544 kb)

## Footnotes

1 Available from www.zackenberg.dk.
