# Supplementary material for: Transitions in high-Arctic vegetation growth patterns and ecosystem productivity tracked with automated cameras from 2000 to 2013
Source: Ambio. 2017 Jan 23;46(Suppl 1):39–52. doi: 10.1007/s13280-016-0864-8 (PMC5258658; doi:10.1007/s13280-016-0864-8)
Supplement: Supplementary file 1 — Supplementary material 1 (PDF 544 kb) [file 13280_2016_864_MOESM1_ESM.pdf]

Ambio

Electronic Supplementary Materials
This supplementary material has not been peer reviewed.

Title: **Transitions in high-Arctic vegetation growth patterns and ecosystem productivity tracked with automated cameras from 2000-2013**

Authors: Andreas Westergaard-Nielsen, Magnus Lund, Stine Højlund Pedersen, Niels Martin Schmidt, Stephen Klosterman, Jakob Abermann, Birger Ulf Hansen

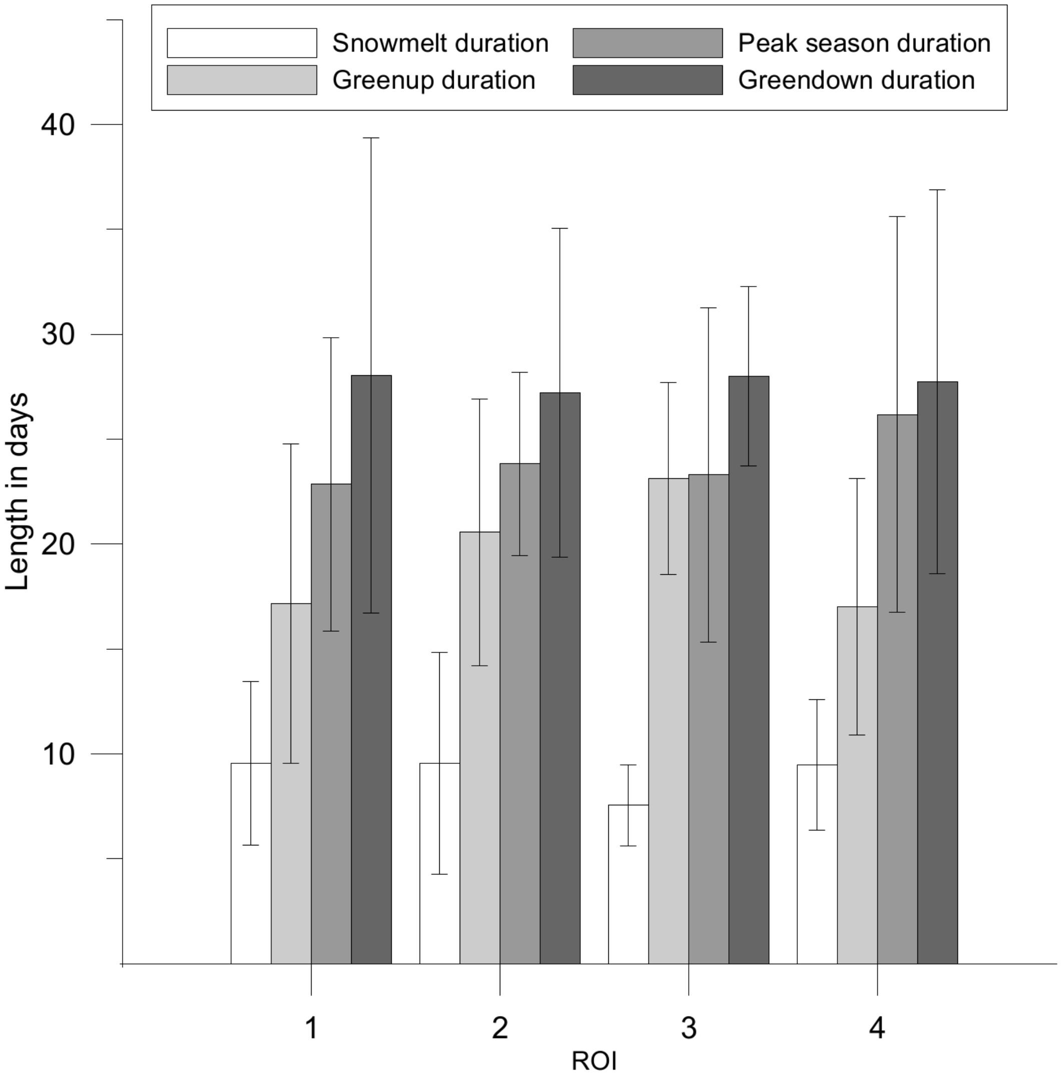

Figure S1: Average durations of snowmelt and phenological transitions. Standard deviations are highest for greenup and greendown durations. ROI refers to region of interest.

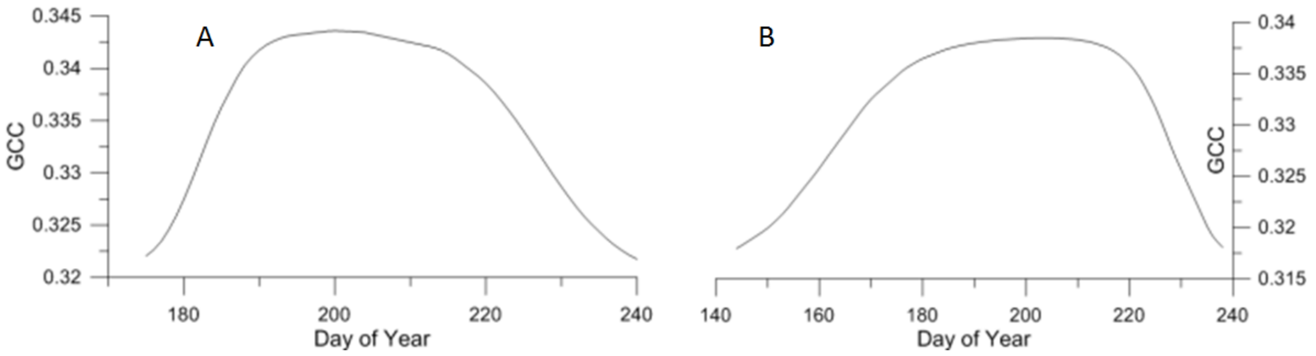

Figure S2: Temporal GCC for (a) snowrich year 2002 and (b) 2009 with limited snowfall, for region 1. Resulting from late end of snowmelt is a fast start of spring rate and slow end of fall rate, while limited snow results in an opposite pattern. The growing season duration is 9 days longer in 2009 based on computed transition dates.

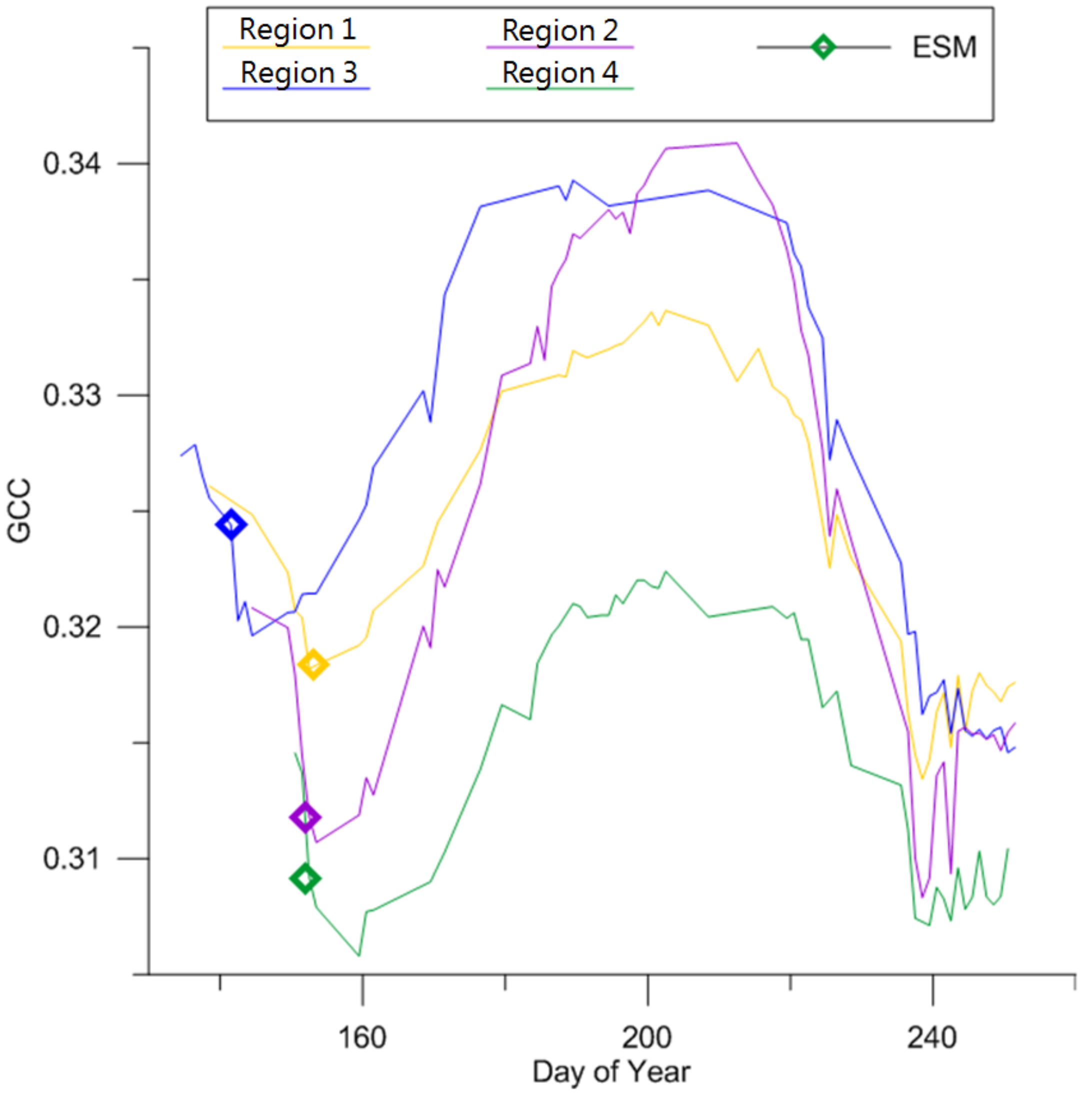

Figure S3: Green Chromatic Coordinate (GCC) for ROI 1-4 in 2009 without a smoothing model fitted. We observed a local minimum following snowmelt. End of snowmelt (ESM) is depicted as symbols in the respective regions.

Table S1: Camera types and matching properties. Due to the oblique angle, the spatial resolution decreases with increasing distance between sensor and object. All images were recorded with auto-settings for white balance, shutter speed, and exposure, as no manual control was possible.

Time period	Cameratype	Resolution in pixels and space
**2000-2005**	1) Kodak DC50	756x504; 1.8-16 m
**2005-2007**	2) Kodak CX6200	1632x1232; 0.8-3.7 m
**2008-2013**	3) HP E427	2848x2136; 0.4-1.9 m

Table S2: Generalized linear model analysis with GPP as response and GCC, Camera model, and the interaction GPP*Camera model as predictors. Low mean square results from the high number of degrees of freedom.

Total R^2 = 0.84, RMSE = 0.0036		
**Correlated variable(s)**	**Mean sq.**	**p-value**
GPP	0.00351	<0.001
Cameratype	0.00076	<0.001
GPP*Cameratype	0.00027	<0.001

Table S3: Dataset with computed transition dates

Year	Region	Start of snowmelt	End of snowmelt	Start of spring	Middle of spring	End of spring	Peak of season	Start of fall	Middle of fall	End of fall
2000	1	140.0	147.0	168.7	178.2	186.9	198.5	213.8	227.5	241.0
2001	1	151.0	164.0	165.7	178.5	191.6	209.5	219.6	235.2	251.3
2002	1	147.5	160.0	173.5	182.1	190.6	199.5	212.6	230.8	248.3
2003	1	144.0	157.0	172.5	182.2	191.7	199.5	204.4	227.5	250.1
2004	1	148.5	155.0	163.0	175.3	187.0	206.5	222.1	235.6	249.3
2005	1	NaN	NaN	NaN	NaN	NaN	NaN	NaN	NaN	NaN
2006	1	164.5	172.0	183.6	186.7	189.8	212.5	220.0	239.4	258.9
2007	1	134.0	153.0	162.4	171.9	181.1	195.5	206.7	227.0	246.7
2008	1	154.0	161.5	163.0	179.8	196.5	210.5	220.7	232.6	244.5
2009	1	136.0	141.5	154.6	165.7	176.8	202.5	218.1	228.6	238.8
2010	1	153.0	161.0	168.3	177.6	187.0	198.5	214.4	229.2	244.1
2011	1	147.5	158.0	163.5	174.9	186.5	202.5	214.0	230.6	247.3
2012	1	163.5	169.0	180.1	189.3	198.7	210.5	222.5	234.9	247.4
2013	1	135.0	143.5	160.6	174.5	188.2	195.5	208.1	219.0	229.9
2000	2	162.5	172.5	177.8	182.6	187.3	200.5	213.0	230.2	248.3
2001	2	156.0	177.5	184.0	188.9	194.6	208.5	222.5	233.3	244.0
2002	2	160.0	179.0	179.3	185.6	192.2	203.5	219.3	238.8	258.4
2003	2	158.5	166.5	173.8	181.1	188.3	194.5	207.6	232.4	257.3
2004	2	160.0	168.0	178.4	185.6	193.1	200.5	221.6	233.8	245.3
2005	2	NaN	NaN	NaN	NaN	NaN	NaN	NaN	NaN	NaN
2006	2	173.5	185.5	179.1	185.4	191.3	213.5	220.6	243.3	265.9
2007	2	158.5	164.0	169.9	180.7	191.2	202.5	205.9	224.0	241.6
2008	2	167.0	178.0	183.9	190.6	197.5	206.5	217.7	232.4	247.0
2009	2	145.0	152.0	163.0	178.2	193.3	209.5	216.2	226.4	236.7
2010	2	162.5	167.5	170.9	180.5	189.8	199.5	216.3	227.0	238.2
2011	2	162.0	168.0	170.7	182.5	194.7	203.5	213.2	225.1	237.0
2012	2	172.5	178.0	187.1	193.6	200.1	206.5	218.1	232.0	246.0
2013	2	146.0	151.5	162.8	177.0	192.6	197.5	203.2	222.9	242.4
2000	3	158.0	167.0	174.9	180.6	186.8	206.5	217.0	227.4	237.7
2001	3	165.0	172.5	183.2	187.8	192.5	202.0	220.7	234.9	248.6
2002	3	160.5	167.0	175.2	182.8	190.4	208.0	213.1	230.0	246.5
2003	3	162.0	168.0	166.3	177.3	188.1	198.5	208.8	230.3	251.8
2004	3	157.5	165.0	177.9	186.2	194.7	206.5	218.7	232.5	246.4
2005	3	NaN	NaN	NaN	NaN	NaN	NaN	NaN	NaN	NaN
2006	3	168.0	176.5	186.6	188.1	189.5	214.5	229.7	246.9	264.1
2007	3	154.5	163.0	166.6	178.9	190.9	202.5	214.2	231.9	249.6
2008	3	163.0	169.0	176.4	186.9	197.3	209.0	221.0	232.0	243.0
2009	3	142.5	153.0	159.8	171.8	183.6	205.9	215.8	226.9	237.5
2010	3	160.5	167.0	169.5	181.0	192.2	200.5	210.5	226.5	242.3

2011	3	161.5	165.5	166.5	181.7	196.8	206.5	218.5	225.9	232.7
2012	3	172.0	178.5	188.3	192.9	197.7	211.5	224.1	233.9	243.7
2013	3	141.0	152.0	153.6	167.3	180.8	193.5	208.3	217.5	226.5
2000	4	160.5	171.5	179.0	182.6	186.0	198.5	219.6	230.0	240.0
2001	4	165.0	177.5	189.9	192.5	195.4	215.5	225.8	237.4	248.4
2002	4	165.5	179.0	183.3	189.4	195.8	203.5	212.1	233.0	255.4
2003	4	163.0	170.5	172.0	179.5	187.2	194.5	214.3	234.6	254.8
2004	4	158.5	169.5	174.6	183.4	192.1	200.5	234.1	237.3	240.4
2005	4	NaN	NaN	NaN	NaN	NaN	NaN	NaN	NaN	NaN
2006	4	176.5	185.5	189.3	NaN	NaN	219.5	212.4	237.8	263.1
2007	4	158.0	165.0	172.2	184.6	196.1	202.5	220.2	231.3	242.8
2008	4	171.0	178.5	186.2	190.0	194.0	205.5	216.6	231.9	246.8
2009	4	142.0	152.0	161.5	175.2	188.9	205.5	220.0	228.8	237.3
2010	4	163.0	169.0	176.8	184.6	192.1	203.5	217.2	229.8	242.5
2011	4	158.0	165.5	167.5	177.8	188.4	205.5	216.3	227.8	239.0
2012	4	176.0	181.0	187.9	193.8	200.0	210.5	225.5	236.7	247.8
2013	4	136.0	151.5	155.6	168.9	182.0	192.5	207.3	222.0	236.4
